# A novel peptide that improves metabolic parameters without adverse central nervous system effects

**DOI:** 10.1038/s41598-017-13690-9

**Published:** 2017-11-01

**Authors:** Patrícia Reckziegel, William T. Festuccia, Luiz R. G. Britto, Karen L. Lopes Jang, Carolina M. Romão, Joel C. Heimann, Manoela V. Fogaça, Naielly S. Rodrigues, Nicole R. Silva, Francisco S. Guimarães, Rosangela A. S. Eichler, Achla Gupta, Ivone Gomes, Lakshmi A. Devi, Andrea S. Heimann, Emer S. Ferro

**Affiliations:** 1 Department of Pharmacology, Biomedical Science Institute, University of São Paulo, São Paulo, 05508-000 SP Brazil; 2 Department of Physiology and Biophysics, Biomedical Science Institute, University of São Paulo, São Paulo, 05508-000 SP Brazil; 3 Department of Internal Medicine, School of Medicine, University of São Paulo, São Paulo, 01246-903 SP Brazil; 4 Department of Pharmacology, Ribeirão Preto Medical School, University of São Paulo, Ribeirão Preto, 14049-900 SP Brazil; 5 Department of Pharmacological Sciences, Icahn School of Medicine at Mount Sinai, New York, 10029 NY USA; 6 Proteimax Biotechnology LTDA, São Paulo, 05581-001 SP Brazil

## Abstract

Intracellular peptides generated by limited proteolysis are likely to function inside and outside cells and could represent new possibilities for drug development. Here, we used several conformational-sensitive antibodies targeting G-protein coupled receptors to screen for novel pharmacological active peptides. We find that one of these peptides, DITADDEPLT activates cannabinoid type 1 receptors. Single amino acid modifications identified a novel peptide, DIIADDEPLT (Pep19), with slightly better inverse agonist activity at cannabinoid type 1 receptors. Pep19 induced uncoupling protein 1 expression in both white adipose tissue and 3T3-L1 differentiated adipocytes; in the latter, Pep19 activates pERK1/2 and AKT signaling pathways. Uncoupling protein 1 expression induced by Pep19 in 3T3-L1 differentiated adipocytes is blocked by AM251, a cannabinoid type 1 receptors antagonist. Oral administration of Pep19 into diet-induced obese *Wistar* rats significantly reduces adiposity index, whole body weight, glucose, triacylglycerol, cholesterol and blood pressure, without altering heart rate; changes in the number and size of adipocytes were also observed. Pep19 has no central nervous system effects as suggested by the lack of brain c-Fos expression, cell toxicity, induction of the cannabinoid tetrad, depressive- and anxiety-like behaviors. Therefore, Pep19 has several advantages over previously identified peripherally active cannabinoid compounds, and could have clinical applications.

## Introduction

Proteasomes play a key role in living organisms in maintaining optimal protein levels and generating functional intracellular peptides. The catalytic activity of proteasomes is present in archaebacteria, whereas the well-known antigen-generating function of proteasomes emerges only in jawed vertebrates^1^. Therefore, proteasomes may have evolved from a pure degradation function in prokaryotes to a protein-processing function in eukaryotes^2^. Intracellular peptides generated by proteasomes that are not related to antigens have been shown to be functional inside and outside cells^3–7^. Hemopressin (HP, PVNFKFLSH^3^) was the first functional intracellular peptide shown to act outside cells as an inverse agonist of cannabinoid type 1 receptors^4^ (CB1R). N-terminal extended versions of HP, named RVD-HP and VD-HP, were later shown to occur naturally in rodent brains and to have agonist activity at CB1R^8–10^. Similar to HP, one peptide (DITADDEPLT^11^; corresponding to residues 9–17 of peptidyl-prolyl cis-trans isomerase A) screened in this study with conformational-sensitive antibodies so as to identify novel pharmacologically active peptides, exhibited inverse agonistic activity at CB1R (Supplemental Table 1). This original DITADDEPLT sequence^11^ was rationally modified to improve CB1R inverse agonist activity, resulting in the peptide sequence DIIADDEPLT (hereafter named “Pep19”; Supplemental Table 1). Pep19 displaces [^3^H]SR141716A binding to membranes of 3T3-L1 cells expressing CB1R with an IC_50_ ~4.9 × 10^−12^ M (Supplemental Fig. 1). In this study we also examined the effect of Pep19 on signaling pathways of 3T3-L1 differentiated adipocytes and on body-weight and metabolic parameters of diet induced obese (DIO) rats. We find that Pep19 ameliorates several metabolic parameters of DIO rats, without detectable central nervous system (CNS) action. The mechanism of action of Pep19 seems to include induction of uncoupling protein 1 (UCP1) expression in adipocytes.

## Results and Discussion

**Figure 1:**
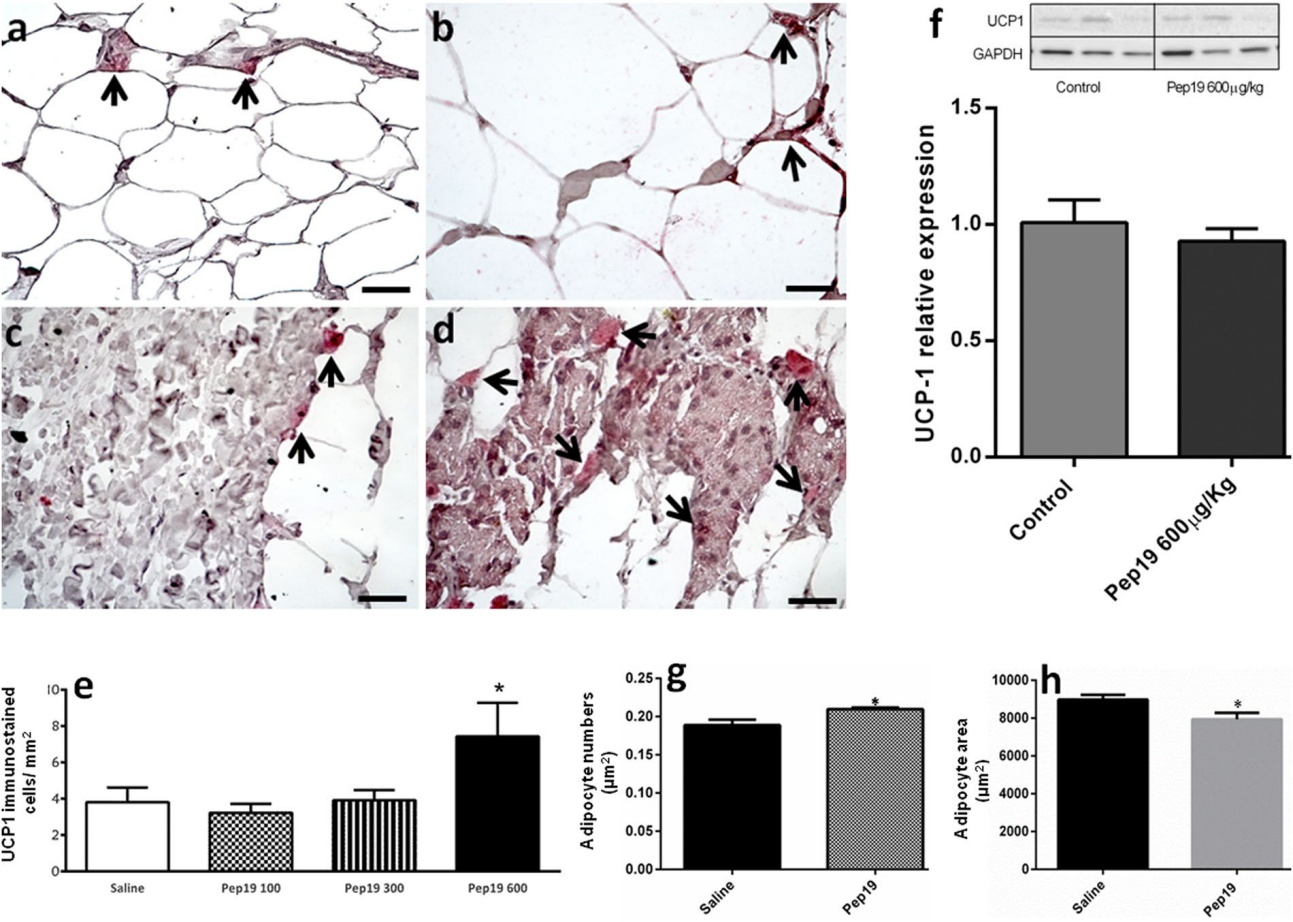
Quantitative analyses in inguinal adipose tissue of DIO rats. Panels a–d: Immunohistochemical representative images (400x magnification, scale bar = 50 µm) showing specific UCP1-immunostained cells from the inguinal white adipose tissue of DIO *Wistar* rats (oral treatments: panel a, saline; panel b, Pep19, 100 μg/Kg; panel c, Pep19, 300 μg/Kg; panel d, Pep19, 600 μg/Kg). Quantitative analyses of UCP1 labeled cells were determined from at least 25 different fields in each slice (n = 8–10), and examined using ImageJ software at 400x magnification; results are expressed as number of UCP1 positive cells per mm^2^. Representative UCP1-immunostained cells (fast red dye) obtained from each treatment are indicated by arrows. Panel e: Quantitative analyses suggest that oral administration of Pep19 600 μg/Kg increases the number of UCP1-immunostained cells in the inguinal adipose tissue compared to saline or other treatments (n = 8–10; *p < 0.05, saline *vs* Pep19 600 μg/Kg). Note that in Pep19 treated animals (panels, c and d) immunostained-UCP1 cells seem to occur in fibrotic-like areas of the adipose tissue. Panel f: Quantitative *Western* blot analysis suggested similar levels of UCP1 expression in either saline or Pep19 (600 μg/Kg) treated groups of DIO animals; (each line shown on the upper panel f, is representative of one individual animal treated with either saline or Pep19 600 μg/Kg; n = 7). Note that the ratio of UCP1 negative cells to positive cells is fairly large and this could contribute to lack in change in the protein levels seen in the Western Blots (panel f). Panel g: Quantitative analyses of the number of adipocytes (adipocytes/µm^2^; 500–800 cells were counted per animal (n = 3), in 14–18 different fields of the H&E stained slices of adipose tissue from animals treated with either saline or Pep19 (600 μg/Kg; *p < 0.05). Panel h: Quantitative analyses of the adipocyte area were performed measuring 15 different adipocytes in each of the 6 fields analyzed per H&E stained slices from each animal (n = 3), using H&E stained slices of adipose tissue from animals treated with either saline or Pep19 (600 μg/Kg; *p < 0.01). All results are expressed as the means ± standard error of the mean (SEM). The statistical comparisons were performed using Student’s t-test or analysis of variance (ANOVA), followed by ad-hoc Tukey’s test using GraphPad Prism software. Crude Western blot membranes are shown on Supplemental material.

Oral administration of Pep19 to DIO *Wistar* rats improved several metabolic parameters, including a reduction in serum glucose, triacylglycerol and blood pressure, without changing heart rate (Table 1). Pep19 reduced the whole adiposity index and the mass of gonadal and mesenteric adipose tissues (Table 1). Oral administration of Pep19 significantly increased the expression of UCP1 in specific cells of the inguinal adipose tissue (Fig. 1a–e), although this was not seen when the overall expression of UCP1 was evaluated by Western blotting (Fig. 1f); these results can be explained by the fact that the difference in UCP1 expression is quite small, and this could contribute to the lack in overall change in signal detected by Western blotting. Next, we analyzed the inguinal adipose tissue of DIO rats treated with Pep19 for changes in the number and size of adipocytes. We find an increase in the number (Fig. 1g) of adipocytes with a significant decrease in their size (Fig. 1h).

**Table 1:** Oral administration of saline, Pep19 or rimonabant to DIO *Wistar* rats.

	Saline	Pep 19 (50 µg/Kg)	Pep 19 (100 µg/Kg)	Pep 19 (300 µg/Kg)	Pep 19 (600 µg/Kg)	Rimonabant (100 µg/Kg)
Body weight (% of control day zero)	108 ± 1.1 (n = 20)	110 ± 1.0 (n = 10)	104 ± 1.7 (n = 20)*	106 ± 0.9 (n = 10)*	108 ± 1.1 (n = 10)	111 ± 1.0 (n = 10)
Food intake (g/day per animal)	13.83 ± 0.5 (n = 10)	ND	13.34 ± 0.9 (n = 10)	12.63 ± 1.1 (n = 10)	12.49 ± 2.3 (n = 10)	ND
Gonadal Adipose Tissue weight (g)	2.12 ± 0.2 (n = 20)	1.82 ± 0.2 (n = 9)	1.58 ± 0.1 (n = 20)*	2.25 ± 0.2 (n = 9)	1.81 ± 0.1 (n = 10)	1.75 ± 0.1 (n = 10)*
Retroperitoneal Adipose Tissue weight (g)	2.15 ± 0.2 (n = 19)	2.19 ± 0.2 (n = 9)	2.6 ± 0.2 (n = 20)	2.76 ± 0.3 (n = 9)	2.47 ± 0.2 (n = 10)	2.10 ± 0.1 (n = 10)
Mesenteric Adipose Tissue weight (g)	1.01 ± 0.1 (n = 18)	0.76 ± 0.1 (n = 7)*	0.80 ± 0.1 (n = 10)*	1.15 ± 0.1 (n = 9)	0.96 ± 0.1 (n = 10)	0.86 ± 0.1 (n = 9)
Adiposity Index (total/100 g)	7.64 ± 0.5 (n = 18)	8.51 ± 0.4 (n = 7)	8.64 ± 0.6 (n = 17)	8.94 ± 0.7 (n = 9)	4.91 ± 0.3 (n = 10)*	5.85 ± 0.2 (n = 9)*
Glucose level (mg/dl)	134.20 ± 3.3 (n = 15)	130.9 ± 4.3 (n = 9)	98.1 ± 2.0 (n = 20)*	102.7 ± 1.8 (n = 9)*	95.0 ± 3.5 (n = 8)**	118.3 ± 4.2(n = 9)*
Insulin (ng/mL)	3.27 ± 0.3 (n = 5)	2.18 ± 0.4 (n = 5)*	2.35 ± 0.2 (n = 5)*	ND	ND	2.71 ± 0.8 (n = 5)
Triacylglycerol (% of control day zero)	98.0 ± 9.5 (n = 18)	132 ± 4.0 (n = 9)*	90.3 ± 9.4 (n = 17)	60.0 ± 6.2 (n = 8)*	57.0 ± 7.1 (n = 9)*	112 ± 7.7 (n = 10)
Cholesterol (% of control day zero)	132.8 ± 18.3 (n = 8)	81.21 ± 5.2 (n = 9)*	92.17 ± 4.4 (n = 10)*	ND	ND	98.51 ± 5.8 (n = 9)*
Blood pressure (mmHg)	129.7 ± 0.8 (n = 10)	ND	116.4 ± 1.5 (n = 10)*	102.7 ± 1.0 (n = 9)*	113.8 ± 0.4 (n = 10)*	ND
Heart rate (beats/min)	387.3 ± 7.6(n = 3)	ND	405.3 ± 2.3 (n = 3)	399.9 ± 5.8 (n = 3)	408.5 ± 4.1 (n = 3)	ND

**Figure 2:**
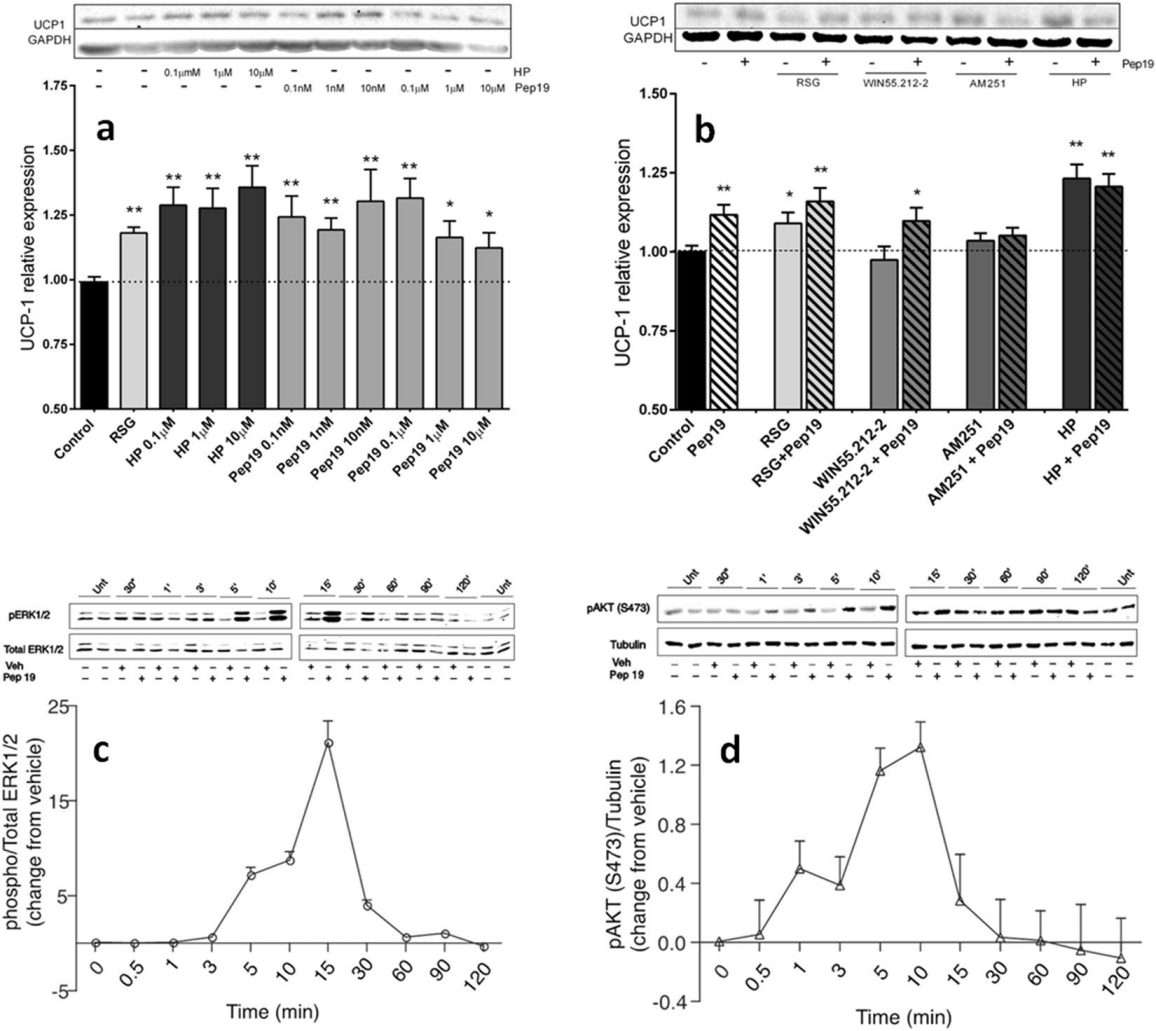
Signaling pathways induced by Pep19 in 3T3-L1 adipocyte cells. Panels a and b: Relative UCP1 expression in 3T3-L1 adipocyte cells. Panel a: Cells were exposed to rosiglitazone (RSG, 5µM), or different concentrations of hemopressin (HP, 0.1–10 µM) or Pep19 (0.1–10 µM). Panel b: 3T3-L1 adipocyte cells exposed to Pep19 (1 µM) for 24 h in the absence or presence of either RSG (5 µM), the CB1R agonist WIN55,212–2 (1 µM), the CB1R antagonist AM251 (1 µM) or the CB1R inverse agonist HP (1 µM). Western blots were conducted using mouse anti-UCP1 antibodies, and anti-GAPDH antibodies were used as loading controls. Panels c and d: 3T3-L1 cells were starved for 16 h in serum-free medium prior to stimulation (vehicle or Pep19, 1 μM) for the indicated time period. Western blots were carried out using: Panel c, mouse monoclonal anti-phosphoERK1/2, and rabbit polyclonal anti-total ERK1/2; Panel d, rabbit phospho-AKT S473 (anti-pAKT, S473) and mouse monoclonal anti-tubulin antibodies. Imaging and band intensity measurements were performed using the Odyssey imaging system (LI-COR, Lincoln, NE) according to the manufacturer’s protocols. Data are representative of three independent experiments that produced similar results. Unt, cells not treated with vehicle (Veh) or peptide 19 (Pep19). The statistical comparisons were performed using Student’s t-test or analysis of variance (ANOVA), followed by ad-hoc Tukey’s test using GraphPad Prism software *p < 0.05; **p < 0.001. Crude Western blot membranes are shown on Supplemental material.

The pharmacological effects of Pep19 on UCP1 protein expression were further investigated in 3T3-L1 adipose cells, to evaluate if Pep19 could be regulating energy metabolism as previously described for endocannabinoids^12–15^. 3T3-L1 is a well-characterized cell model for studying the adipose tissue metabolism^16–18^. Rosiglitazone (RSG), used as a positive control for UCP1 activation, is an agonist for peroxisome proliferator-activated receptor-γ (PPAR-γ), a nuclear receptor highly expressed in brown and white adipose tissues that acts as a master transcriptional regulator of brown adipocyte differentiation required for tissue development, function and survival^19–23^. Pep19 and HP similar to RSG increased the expression of UCP1 after 24 h treatment in 3T3-L1 adipose cells (Fig. 2a). The CB1R antagonist AM251, but not the agonist WIN55,212–2, prevented UCP1 activation by Pep19 (Fig. 2b). Pep19 was observed to transiently activate pERK1/2 and AKT phosphorylation in 3T3-L1 cells (Fig. 2c and d). These data suggest that Pep19 could trigger UCP1 increased expression through activation of AKT and pERK1/2 pathways, which are known to induce the expression of PPARγ coactivator 1α^24,25^. Further studies are necessary to investigate the relationship between pERK1/2, pAKT and UCP1 activation and the metabolic improvements of DIO rats orally administrated with Pep19.

**Figure 3:**
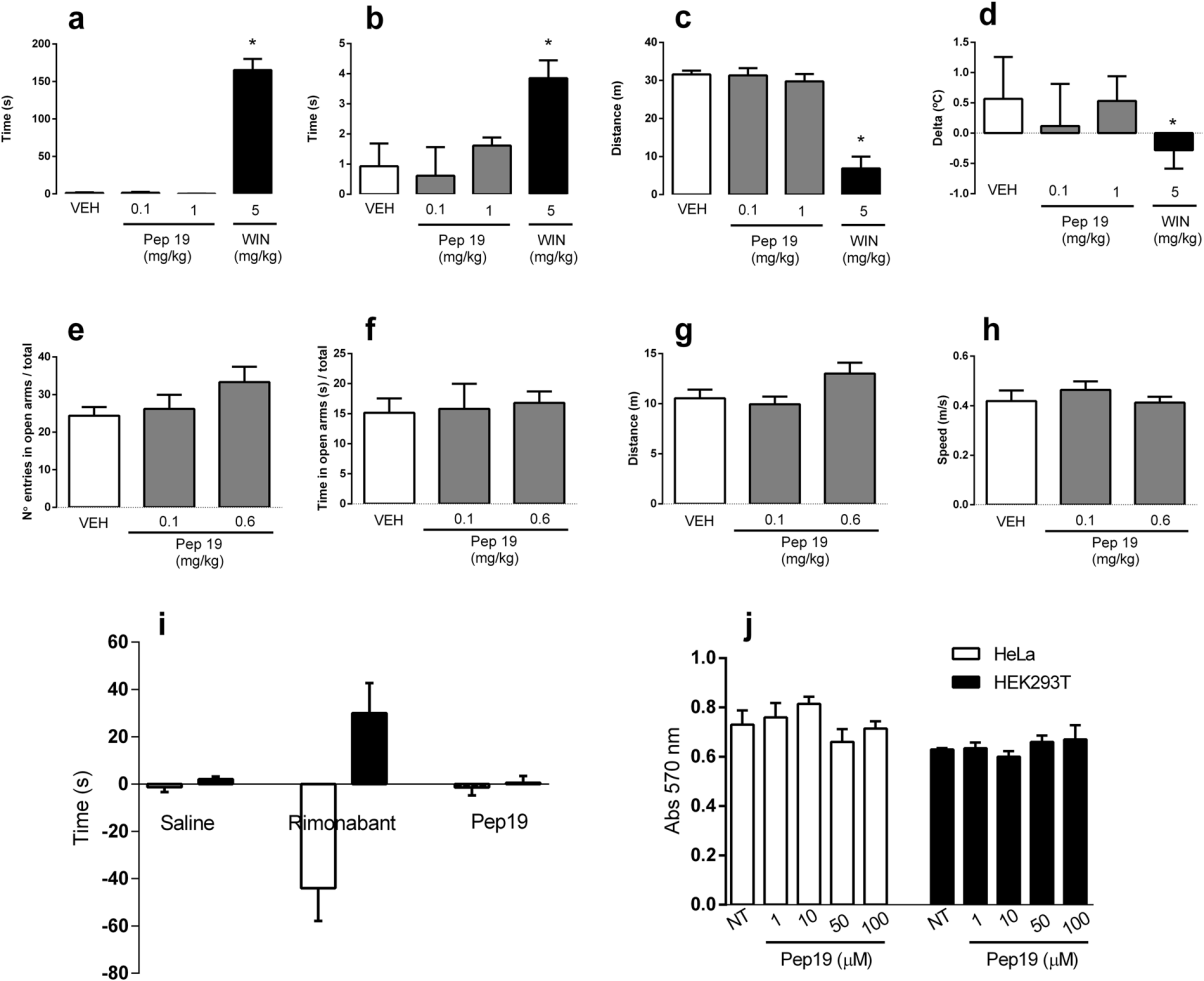
Several *in vivo* assays suggest that Pep19 lacks typical cannabinoid undesired CNS effects. Tetrad cannabinoid responses comprising catalepsy, hypothermia, hypolocomotion and analgesia were evaluated and are shown in **(a)** catalepsy, **(b)** hot plate test, **(c)** locomotor activity and **(d)** tail temperature. Note that no significant differences were observed between the groups treated with vehicle or Pep19 (one-way ANOVA followed by Tukey’s test, n = 6/group), whereas the CB1R agonist WIN 55,212-12 affected all the parameters evaluated (a–d; *p < 0.05). In the elevated plus maze **(e**,**f)** the anxiogenic-like activity of Pep19 was evaluated following oral administration (100–600 µg/kg). Note that no significant differences were observed between the groups treated with vehicle or Pep19 (one-way ANOVA followed by Tukey’s test, n = 7–9/group). In addition, no significant differences were observed between experimental groups in locomotor activity **(g)** and speed travelled **(h)** in the elevated plus maze. In the forced swim test **(i)**, Pep19 produced no significant differences compared with (one-way ANOVA followed by Tukey’s test, n = 6/group), whereas the CB1R antagonist rimonabant affected both time spent swimming (empty bars) and time spent floating (full bars) (i; n = 8–10/group; *p < 0.05). The MTT (3-(4,5-dimethylthiazol-2-yl)-2,5-diphenyltetrazolium bromide) tetrazolium) reduction assays **(j)** were conducted in both HeLa and HEK293T cells, suggest that Pep19 (up to 100 µM) has no ability to induce cell toxicity (j; n = 3/group). In MTT tests for the positive control of 100% permeabilized cells they were treated with 50% ethanol for 1 min and then stained as described above (data not shown). Each bar represents the mean ± SEM.

The endocannabinoid system is over activated in overweight and obese humans^26^ making antagonists or inverse agonists of CB1R an attractive strategy to treat obesity. However, undesired CNS effects of CB1R antagonists need to be avoided to prevent anxiety- and depressive-like responses^27^. The CNS effects of Pep19 were extensively investigated at behavioral and biochemical levels. In rodents, CNS activation of CB1R induces a tetrad of behavioral responses comprising catalepsy, hypothermia, analgesia and hypolocomotion^28^. Thus we evaluated whether administration of Pep19 produced these behavioral changes (Fig. 3a–d). We find that, in contrast to the positive control WIN 55,212-2 (a CB1R agonist) Pep19 has no effect on the cannabinoid tetrad (Fig. 3a–d). In addition, depressive and anxiety-like behaviors were also investigated in rats treated with Pep19 using the forced swim and plus-maze tests, respectively (Fig. 3e–i). Pep19 did not alter distance moved, speed, and the ratio of open/total arms entries and time spent in the plus-maze test, nor swim and/or floating times in the forced swim test, compared to control animals (Fig. 3e–i). We find that Pep19 does not exhibit toxic activity in both HEK293 and HeLa cell lines (Fig. 3j). Additional animal behavior assays suggest that even after 10 days of chronic treatment with Pep19 (0.1 or 1 mg/kg, i.p.), no typical cannabinoid undesired CNS effects were observed (Supplemental Fig. 2). Taken together, these results suggest that Pep19 does not exhibit CNS activity or cellular toxicity.

**Figure 4:**
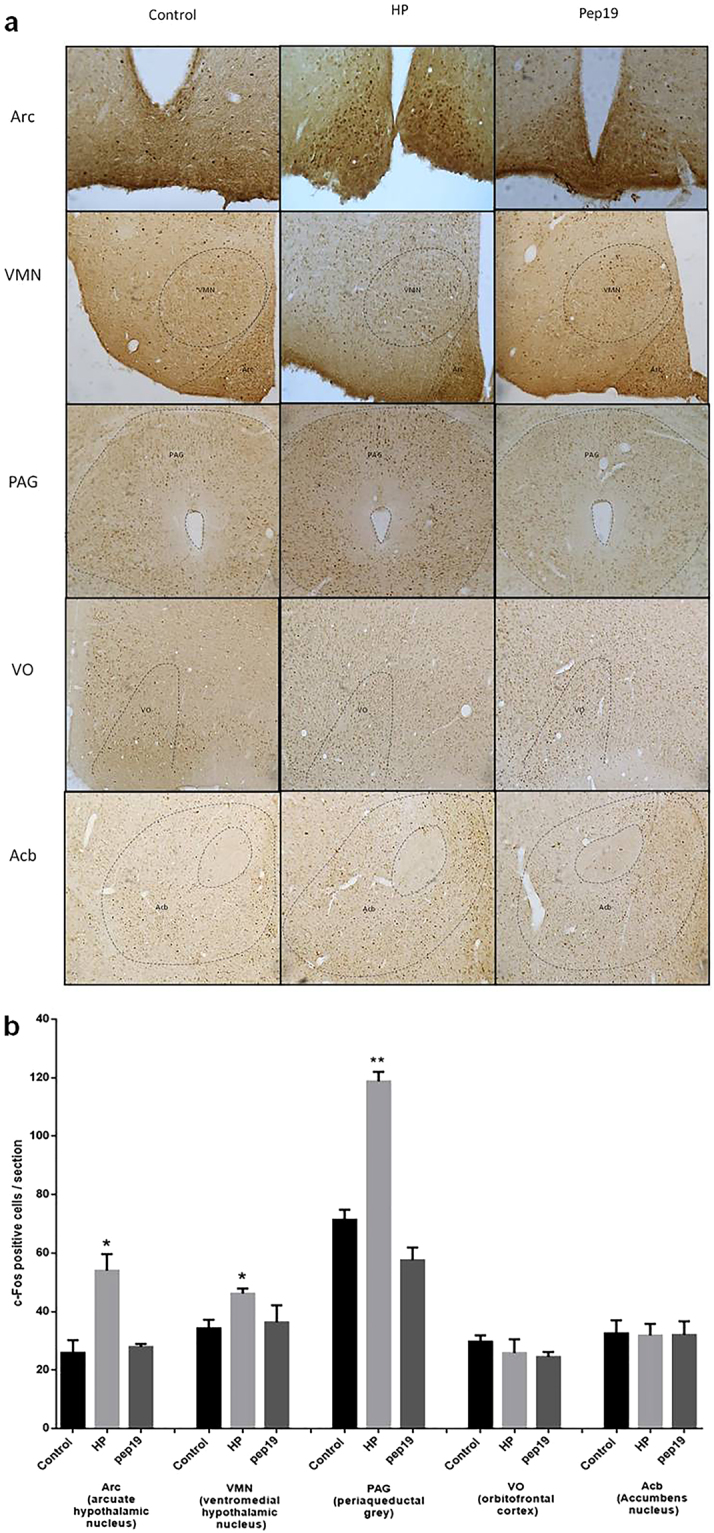
cFos immunohistochemistry in brain regions of mice treated with vehicle, Pep19 or hemopressin (HP). Panel a: pictures are representatives of brain regions such as arcuate hypothalamic nucleus (Arc), ventromedial hypothalamic nucleus (VMN), periaqueductal grey (PAG), orbitofrontal cortex (VO) or accumbens nucleus (Acb). Panel b: The numbers of cFos immunoreactive cells, in each of the indicated brain areas were analyzed using Image J software. Results are presented as mean ± SEM (n = 3/group). The respective pharmacological treatment (Pep19 or HP, 1 mg/kg administrated orally) was compared with control (vehicle, water) by Student’s t-test or analysis of variance (ANOVA), followed by ad-hoc Tukey’s test (n = 7): *p* < 0.05 (*) or *p* < 0.02 (**). Note that Pep19 treatment did not increase the number of cFos positive cells in any of the evaluated areas, this is different from HP treatment that increased the number of cFos positive cells in Arc, VMN and PAG.

To further investigate the behavioral results at a biochemical level, c-Fos activation was investigated in the brains of mice treated with Pep19, and compared to HP treatment (Fig. 4). CB1R is widely distributed throughout the brain, including CNS areas that are involved in the regulation of food intake and metabolism (for review, see^29^). In agreement with behavioral tests, c-Fos immunohistochemistry of Pep19 treated mice did not show activation of CNS areas frequently related to the regulation of energy homeostasis, reward processes and pain, such as arcuate hypothalamic nucleus (Arc), ventromedial hypothalamic nucleus (VMN), periaqueductal grey (PAG), orbitofrontal cortex (VO), and accumbens nucleus (Acb). The CB1R inverse agonist HP activated Arc, VMN and PAG regions, in agreement with a previous study^30^ and with its antinociceptive activity^31^. These results further suggest a lack of CNS activity for Pep19.

In this study, Pep19 was selected from a list of thirty two distinctive intracellular peptides due to its activity at CB1R using a method previously used to characterize HP peptides^4^. HP and its naturally-occurring extended versions VD-HP and RVD-HP among others^8,9^ were the only peptides described to act on cannabinoid receptors^4,10,32^. Pep19 seems to work similarly to HP, reducing body weight and improving metabolic parameters. However, Pep19 has the advantage of lacking CNS activity; therefore, Pep19 has no undesired side effects associated with CB1R inverse agonists such as rimonabant and HP. Pep19 activates UCP1 expression on 3T3-L1 adipocyte cells and on specific cells of white adipose tissue of DIO *Wistar* rats.

Thus, Pep19 has unexpected pharmacological activity acting both as an inverse agonist and as an agonist of cannabinoid receptors, or might be processed to a form that has agonist activity in cells and/or has a biased agonistic activity. These results resemble the cannabinoid activity of HP, which is well documented to bind and signal as an inverse agonist/negative allosteric modulator of CB1R and to have agonistic-like antinociceptive action *in vivo* ^4,28^. A possible explanation for these paradoxical effects could be that endocannabinoids activate distinct signal transduction pathways under different paradigms (*i*.*e*. cross talk), while measuring distinctive parameters, leading to Pep19 observed effects herein.

Another surprising finding of the present study is that Pep19 is orally active. A number of previous reports also described orally active peptides including HPs and peptides C111/C112 from bonito liver^31,32^ and peptides IPP and VPP in addition to tryptic peptides from casein^33–37^. Possibly a similar mechanism is involved in maintaining the biological activity of Pep19 after oral administration, although not much is known about it at this time. It is also possible that Pep19 is processed to shorter bioactive peptides, some of which may be responsible for additional biological activity. Therefore, further investigations are necessary to better clarify the mechanism of action of Pep19 on improving metabolic parameters.

In this study, we demonstrate that oral treatment of DIO *Wistar* rats with Pep19 reduces adiposity index and body weight and improves several metabolic parameters including a reduction in the glucose, triacylglycerol, cholesterol and blood pressure, without altering heart rate. Pep19 does not exhibit undesired CNS effects in rodents as observed here in several behavior and cellular toxicity models. Taken together, our results indicate that Pep19 has several advantages over previously identified peripherally active cannabinoids and therefore could be a novel pharmacological tool for preventing or treating obesity and metabolic disorders.

## Methods

### Peptides and conformational-sensitive antibodies

All peptides and conformational-sensitive antibodies were from Proteimax Biotechnology LTDA, São Paulo, SP, Brazil.

### Animals

The animal experiments were conducted with male *Wistar* rats or C57BL/6J mice. Animals were kept with free access to food and water in a room with controlled temperature (22–23 °C) and in 12 h light/dark cycles with lights on at 6:00 am. The number of animals used was the minimum necessary to obtain statistically significant results and they were maintained and used in accordance to the guidelines of the National Council for Control of Animal Experiments (CONCEA), following international norms of animal care and maintenance. Thus, we hereby state that all experimental protocols were previously approved by University of São Paulo Ethic committee councils from Biomedical Science Institute (approval number for mice experimentation ICB/USP 43/2015) and Medical School (approval number for rats experimentation FMUSP/CAPPesq 0550/07).

### Agonist-mediated change in recognition by the conformational sensitive-antibodies

Effect of peptide ligand treatment on receptor recognition by the antibodies was assayed using an ELISA based assay, as previously described^38^. Briefly, 5 µg/well of tissue membranes were added to each well of a 96 well plate (high binding microplate, Greiner Bio-One, USA) and incubated at room temperature overnight. The next day, wells were washed with phosphate-buffered saline (PBS) and incubated without or with 1 µM of peptide ligands (Supplemental Table 1) in 50 mM Tris-Cl, pH 7.5, for 30 min at 37 °C (in the presence of a protease inhibitor mixture; Sigma, USA). Wells were quickly rinsed three times (within 5 min) with cold PBS and membranes fixed with 3.7% formaldehyde in 1X PBS for 20 min at room temperature, and then washed 5 times with PBS. ELISA was carried out by incubating membranes with 1% bovine serum albumin (BSA) and 5% sucrose in PBS for 1 h at 37 °C, followed by overnight incubation at 4 °C with a 1:500 dilution of affinity purified primary antiserum in 1% BSA in PBS. The wells were then washed three times with PBS (5 min each wash) followed by a 1-h incubation at 37 °C with 1:500 dilution (in PBS) of secondary anti-rabbit antibodies coupled to horseradish peroxidase. The wells were washed three times with PBS (5 min each wash), and color was developed by the addition of the substrate, o-phenylenediamine (5 mg/10 mL in 0.15 M citrate buffer, pH 5, containing 15 μl of H_2_O_2_). Absorbance at 490 nm was measured with a Bio-Rad ELISA reader. The absorbance obtained from membranes incubated without ligands only in 50 mM Tris-Cl, pH 7.5, for 30 min at 37 °C were considered as 100% of absorbance. These results were compared to absorbance values obtained in the presence of each of the original peptide ligand (Supplementary Table 1). All results are expressed as the means ± SEM. The statistical comparisons were performed using t-test analysis.

### Diet induced obesity

For the diet-induced obesity (DIO) model, male *Wistar* rats received instead of water a 20% sucrose solution for 13 consecutive weeks. All animals including controls received food *ad libitum*. Pep19 was administrated orally by gavages. After 13 weeks of treatment all rats were sacrificed by decapitation (after overnight fasting). Blood from the trunk was collected into tubes without anticoagulants and immediately centrifuged (3000 x g for 15 min at 4 °C), and serum was stored at −20 °C. Blood serum was used for all biochemical determinations. The adipose tissues masses were used to calculate the adiposity index (the percentage of the sum of all white adipose tissue pads in relation to total body weight).

### Biochemical assays

Serum cholesterol and triglycerides were quantified enzymatically using commercial kits from Labtest Diagnóstica (Triglicerides Liquiform andLiquiform, Lagoa Santa, MG, Brazil). Insulin levels were determined using specific rat radioimmunoassay kits (Millipore Corporation, Billerica, MA, USA). Blood glucose concentration was measured by a glucometer (Advantage, Roche Diagnostics Corporation, Indianapolis, USA).

### Tail-cuff blood pressure

Measurements of systolic blood pressure were performed at the proximal part of the tail, by a tail-cuff method using the Kent RTBP2000 system and the Kent RTB001-R data acquisition system (Kent Scientific Corporation, Torrington, CT, USA) from 9 to 11 a.m. Before measurements, rats were warmed for 10 min and three stable consecutive measurements were taken and averaged after animals have been trained for proper adaptation to the procedures.

### Adipose tissue haematoxylin and eosin (H&E) staining, UCP-1 immunohistochemistry and Western blot assays

The inguinal white adipose tissue (n = 8–10 per group) from left and right sides were dissected, immediately frozen in liquid nitrogen and stored separately at −80 °C. The inguinal white adipose tissue from one side fromeach animal was thawed in 0.9% saline, fixed in Dubosq-Brazil solution for 1 h, then in 10% formalin phosphate buffer for 24 h. These fragment tissues were included in paraffin blocks by an automated process (Jung-Histokinette 2000, Leica, Nussloch, Dutch). Haematoxylin and eosin (H&E) staining was carried out on paraffin sections (2–3 tissue slices per glass slide) using standard methods. Immunostaining was conducted in five µm thick sections that were deparaffinized, hydrated and incubated with sodium citrate 0,01 M, pH 6 for antigenic retrieval in a hot chamber at 98 °C. Then, the tissue slices (2–3 tissue slices per glass slide) were incubated at 4 °C overnight with anti-UCP1 (Abcam ab10983, San Francisco, CA, USA) primary antibodies (1:800 dilution). After rinsing in PBS for several times to remove the excess of primary antiserum, sections were incubated for 45 min at room temperature with biotinylated anti-rabbit IgG (Vector Labs, Burlingame, CA, USA), followed by a streptavidin-biotin alkaline phosphatase complex (AK-5001, Vector Labs, Burlingame, CA, USA) for 30 min, and finally developed with fast red dye solution (Sigma-Aldrich, Saint Louis, MO, USA). All slices were counterstained with Mayer´s hemalaum and covered with a glycerin-gelatin mixture. The negative controls were performed either omitting the primary antibody or using a preimmune serum; in both cases the results were lack of staining (data not shown). The photomicrographs were taken from different magnifications using a Nikon Eclipse E800 microscope equipped with digital imaging accessories and an epi-fluorescence module (Nikon, Tokyo, Japan). For quantitative analyses of UCP1 labeled cells, from each slice at least 25 different fields were examined in ImageJ software at 400x magnification, and results were expressed as number of UCP1 positive cells per field.

The remaining contralateral side of the inguinal white adipose tissue from DIO *Wistar* rats was used for Western-blot assays. Tissues were homogenized in 1:5 (weight of tissue: volume of buffer) with buffer containing 0.5 mM EGTA, 1% Triton X-100, 0.1% sodium deoxycholate, 0.1% SDS, 140 mM NaCl, and 1 mM PMSF added just before use (Ripa Buffer), centrifuged and the intermediate phase added to Laemmli buffer and heated 95 °C during 5 min. Protein samples were separated on a 12% polyacrylamide gel electrophoresis containing sodium dodecyl sulfate (SDS-PAGE), followed by transfer to polyvinylidene fluoride (PVDF) membranes (Merck Millipore, Darmstadt, Germany). Membranes were blocked for 1 h at room temperature in Tris-HCl-buffered saline, pH 7.4, containing Tween^®^20 0.05% and 5% of nonfat dry milk. Next, membranes were incubated overnight at 4 °C with specific mouse primary antibodies anti-UCP1 (Abcam, ab23841) or GAPDH (sc-32233, Santa Cruz Biotechnology, Dallas, TX, USA) diluted 1:1000 in Tris-HCl-buffered saline, pH 7.4, containning 5% nonfat dry milk. Blots were incubated with horseradish peroxidase-conjugated secondary antibodies for 3 h at room temperature. Western blot bands were visualized with Super-Signal West Pico Chemiluminescent substrate (Thermo Scientific) using the ChemiDoc™ MP Imaging System (BioRad, Hercules, California, USA), and quantified using ImageJ 1.49 software. All results are expressed as the means ± standard error of the mean (SEM). The statistical comparisons were performed using Student’s t-test or analysis of variance (ANOVA), followed by ad-hoc Tukey’s test (n = 7). Data were statistically analyzed with GraphPad Prism software (GraphPad Software Inc, San Diego, CA, USA).

### Quantifying size and number of adipocytes in adipose tissue

The inguinal white adipose tissue stained with H&E as described above was used for quantifying the number and size of adipocytes, as previously described^39^. Briefly, to estimate the number of adipocytes five images from each H&E stained slice per animal were collected at 40x magnification using a Nikon Eclipse E800 microscope equipped with digital imaging accessories. Using ImageJ software, a rectangle of 1 mm^2^ was extracted from every image and the number of cells found in this area was determined; fibrotic areas were avoided. Results are expressed in number of adipocytes/µm^2^ and values were obtained from 500–800 cells counted per slice/animal, in 14–18 different fields of the H&E stained slices. The quantitative analyses of the adipocytes area in µm^2^ were also performed using ImageJ. Fifteen different adipocytes were measure in each of the 6 fields analyzed per slice/animal in H&E stained slices. All results are expressed as the means ± standard error of the mean (SEM). The statistical comparisons were performed using Student’s t-test or analysis of variance (ANOVA), followed by ad-hoc Tukey’s test. Probability less than 0.05 was considered as statistically significant (*p* < 0.05). Data were statistically analyzed with GraphPad Prism software (GraphPad Software Inc, San Diego, CA, USA).

### Tetrad cannabinoid tests

C57BL/6J mice were treated i.p. with Pep19 (0.1 or 1 mg/kg), the CB1R agonist WIN55,212-2 (5 mg/kg) or vehicle (n = 6 per group) and evaluated in the tetrad cannabinoid tests. Another group of animals received Pep19 (0.1 or 1 mg/kg) or saline i.p. for 10 days, once a day. In the last day of treatment the animals were subjected to the tetrad cannabinoid tests 30 minutes after the i.p. administration. The CB1 agonist WIN55,212-2 (5 mg/kg) was administrated i.p. 30 minutes before the experiments as a positive control (n = 6 per group).

#### Catalepsy

It was assayed by the bar test^40^, 30 min after drug administration. Briefly, each mouse was placed on a bar oriented parallel to and approximately 4 cm above the bench surface. Cataleptic behavior was defined as the time the animal remained motionless holding on to the bar; cutoff = 300 s^41^.

#### Tail temperature

It was determined by using a thermal probe (Thermocom®, Nashua, NH 03062, EUA) positioned 50 cm of the animal. The mean of five pictures of five different tail regions was compared between a basal evaluation and another observation 35 min after drug administration.

#### Locomotor activity

It was determined by an open field test, in which a mouse was placed in a circular cage (40 cm diameter), 37 min after drug administration. The distance traveled by the animal was recorded for 10 min by the software ANY-MAZE (Stoelting).

#### Hot plate

The mouse was placed on a heated plate at 56 °C. An experimenter measured the time it took for the mouse to lick the paws or jump off of the hot plate; cutoff = 30 s^42^. The variation between a basal evaluation and another one taken 45 min after drug administration was defined as the result.

### Elevated plus maze

Male *Wistar* rats were tested in a wooden-made elevated plus maze according to Hefner and Holmes^43^ one hour after vehicle or Pep19 i.p. administration (100–600 µg/kg). The percentage of entries, time spent in the open arms, and the number of enclosed arms entries for 5 min were analyzed by the Anymaze Software (version 4.5, Stoelting, USA). Locomotor activity and speed traveled were also recorded.

### Forced swim test

The forced swim test was based on previous designs^44^. Male *Wistar* rats were placed in a cylindrical plexiglass tank (50 cm high and 30 cm diameter) filled with water (19 °C) up to a level of 25 cm from the bottom. The animal’s behavior during the first 5 min of the swim test was scored by a trained observer and the following measures were taken: (1) time spent swimming, defined as movements of all four limbs, swimming around the tank or diving and (2) time spent floating, defined as the time the animal remained immobile with only occasional slight movements to keep the body balanced and the nose above the water.

### Elevated plus maze and forced swim test following chronic treatment with Pep19

Male C57BL/6J mice received a chronic treatment with Pep19 (0.1 or 1 mg/kg) or saline administrated i.p. for 10 days, once a day. The same animal was tested in the EPM and FST 30 and 35 minutes after the last injection, respectively.

### Mice brain *c-Fos* protein immunohistochemistry

Mice C57BL/6J (25 ± 2 g; n = 3 per group) were assigned randomly to receive gavage (v.o.) of either vehicle (water), or 1 mg/kg body weight HP or 1 mg/kg Pep19. All solutions were prepared minutes before use. Two hours post administrations mice were deeply anaesthetized with ketamine and xylazine (i.p.; 1 g/Kg and 0.2 g/kg, respectively) and perfused transcardially with heparinised saline (5000 UI/kg) in phosphate buffer (PB 0.1 M, pH 7.3) followed by 4% paraformaldehyde in PB 0.1 M. The brains were post fixed and kept in 30% sucrose in 0.1 M PB to cryoprotect the tissue. Seven days later, brains were cut in serial coronal sections (30 µm) on dry ice using a sliding microtome (Leica SM 2000R – Nussloch, Germany). The slices were equally divided into 6 wells according to the region from where they were obtained, and the amount of one well was used for c-Fos immunohistochemistry. Sections were stored in PBS 0.1 M at 4 °C until use. Slices were incubated overnight at room temperature in rabbit anti-c-Fos antibody (Santa Cruz Biotechnology, SC-52) diluted to 1:1000 in blocking buffer [normal goat serum (NGS, Jackson Immuno, 005-000-121) in 0.3% Triton X-100 in PB 0.1 M. After washing, the sections were incubated at room temperature for two hours in goat anti-rabbit IgG biotin complex (Jackson Immuno, 111-065-003) diluted 1:200 in 0,3% Triton X-100 in PB 0.1 M, followed by avidin biotin peroxidase complex (Vector Laboratories, PK-6100) diluted in NaCl 0.4 M with 0.3% Triton X-100, two hours at room temperature and, finally, visualized with nickel-intensified diaminobenzidine. Slices were organized in glass slides, dryed and covered with Permount and coverslips.

The regions of interest were identified based on a stereotaxic atlas^45^: arcuate hypothalamic nucleus (Arc), ventromedial hypothalamic nucleus (VMN), periaqueductal grey (PAG), orbitofrontal cortex (VO), accumbens nucleus (Acb). The corresponding images were captured using a Nikon Eclipse E800, camera DC-F1 with Nis Element program. cFos positive cells per section were counted manually using ImageJ (NIH/USA). Results are are expressed as the means ± standard error of the mean (SEM) of c-Fos immunoreactive cells per section in each brain area (5 sections/brain/structure). Each individual pharmacological treatment (Pep19 or HP) was compared with vehicle using Student’s t-test or analysis of variance (ANOVA), followed by ad-hoc Tukey’s test (n = 7). Data were statistically analyzed with GraphPad Prism software (GraphPad Software Inc, San Diego, CA, USA).

### Cell culture and assays

#### HeLa and HEK293-T cells

HeLa and HEK293-T cells were cultured in DMEM (Dulbecco’s Modified Eagle Medium, Gibco®, USA) containing 10% fetal bovine serum (complete medium), penicillin and streptomycin (Gibco®, USA) at 37 °C and in presence of 5% of CO_2_. For cell counting, single cell suspension of HeLa and HEK293-T cells were rinsed with PBS and treated with 0.4% Trypan blue in PBS for 90 s. Excess dye was removed, and the cells were washed twice with PBS before counting. For a control of 100% permeabilized cells they were treated with 50% ethanol for 1 min and then stained as described above. In all of the experiments cellular viability was greater than 98% (*data not shown*). The MTT (3-(4,5-dimethylthiazol-2-yl)-2,5-diphenyltetrazolium bromide) tetrazolium reduction assays were conducted in a 96-well format^46^. The optical density was measured at 570 nm using a SpectraMax M2 (Molecular Devices, Sunnyvale, CA, USA). Three independent experiments were conducted in triplicates for each treatment.

#### 3T3-L1 cells

3T3-L1 pre-adipocytes (American Type Culture Collection, Rockville, MD, USA) were cultured in complete medium comprising of DMEM (Dulbecco’s Modified Eagle Medium), containing 10% fetal bovine serum (Life Technologies, Carlsbad, CA, USA), 0.11 mg/mL pyruvate, 0.025 g/L penicillin (Sigma-Aldrich, Saint Louis, MO, USA) and 0.1 g/L streptomycin (Life Technologies, Carlsbad, CA, USA) at 37 °C and 5% CO_2_. For the experiments analyzing UCP1 expression, 3T3-L1 cells were seeded in 12-well plates at a confluence of 10 × 10^4^ cells/mL/well. One day later, cells were differentiated using normal culture medium supplemented with 500 μM isobutylmethylxanthine (Sigma, St. Louis, MO, USA), 1 μM dexamethasone (Sigma, St. Louis, MO, USA), and 0.85 μg/mL bovine insulin (Sigma, St. Louis, MO, USA). After three days, the differentiation medium was changed to normal culture medium supplemented with 0.85 μg/mL bovine insulin. The fresh medium was renewed every 2 days. After 12 days of differentiation, cells were exposed for 24 h with either 5 µM rosiglitazone (RSG, Sigma, St. Louis, MO, USA), HP or Pep19 at different concentrations or with vehicle (normal culture medium plus protease inhibitor cocktail (Roche, Germany). Subsequent experiments were performed after preincubation for 2 min with and in the absence of either 5 µM rosiglitazone, 1 µM WIN212,12-2, 1 µM AM251, 1 µM HP or vehicle (normal culture medium plus protease inhibitor cocktail (Roche, Germany) with subsequent addition of 1 µM Pep19 in medium. For Western-blot assays, cells were washed twice with ice-cold PBS, the supernatant was removed, and cells were lysed with 40 µL Laemmli buffer, transfered to tubes, sonicated and heated 95 °C during 5 min. Samples were kept at −20 °C till use. Before using, samples were thawed on ice, centrifuged, and proteins were separated on a 12% SDS-PAGE, followed by transfer to PVDF membranes (Merck Millipore, Darmstadt, Germany). Membranes were blocked for 1 h at room temperature in Tris-buffered saline Tween-20 buffer with 5% nonfat dry milk, followed by overnight incubation at 4 °C with specific mouse primary antibodies for UCP1 (Abcam, ab23841) or GAPDH (sc-32233, Santa Cruz Biotechnology, Dallas, TX, USA) diluted 1:1000 in Tris-buffered saline with 5% nonfat dry milk. Blots were incubated with horseradish peroxidase-conjugated secondary antibodies for 3 h at room temperature. Bands were visualized with Super-Signal West Pico Chemiluminescent substrate (Thermo Scientific) and quantified using ImageJ 1.49.

For experiments analyzing phospho/total ERK1/2 and phosphoAKT/tubulin expression, 3T3-L1 cells were seeded on 24-well plates in complete media. Next day, the cells were starved for 16 h in serum-free medium prior to stimulation. Cells were treated with vehicle or Pep19 (1 μM) for different periods of time (1–120 min). Cells were solubilized by directly adding 1 × SDS buffer pre-warmed to 65 °C, followed by sonication with a micro-tip for 5 sec. Proteins were separated by 10% SDS-PAGE and transferred to nitrocellulose membranes for immunoblotting with mouse monoclonal anti-phospho ERK1/2 (anti-pERK, 1:1000; Cell Signaling, USA), and rabbit polyclonal anti-total ERK1/2 (anti-total ERK, 1:1000; Cell Signaling, USA) antibodies. Blots were also treated with rabbit anti-phosphoAKT (S473) (anti-pAKT,1:1000; Cell Signaling, USA) and mouse monoclonal anti-tubulin (1:50,000; Sigma-Aldrich, USA) antibodies. The following antibodies were used as secondary antibodies: IRDye 680-labeled anti-rabbit diluted at 1∶10,000 (Cell Signaling, USA) and IRDye 800-labeled anti-mouse diluted at 1∶10,000 (Cell Signaling, USA). Blotting, imaging and band intensity measurements were performed using the Odyssey imaging system (LI-COR, Lincoln, NE) according to the manufacturer’s protocols.

### Ligand binding studies

Membranes were prepared from 3T3-L1 cells endogenously expressing CB1R, and displacement ligand binding assays were carried out as previously described^8^ with minor modifications. Briefly, membranes (250 μg) were incubated with [^3^H] SR141716A (3 nM) in 50 mM Tris-Cl, pH 7.8, containing 1 mM EGTA, 5 mM MgCl_2_, and protease inhibitor cocktail in the presence of Pep19 (0–10 μM) for 1 h at 30 °C. At the end of the incubation period, membranes were filtered using a Brandel filtration system and GF/B filters (presoaked for 2 h at room temperature with 0.1% polyethyleneimine containing 0.2% fatty acid free BSA), filters were washed 3 times with ice-cold 50 mM Tris-Cl (pH 7.4) and bound radioactivity measured using a liquid scintillation counter.

### Statistical analyses

All results are expressed as the means ± standard error of the mean (SEM). The statistical comparisons were performed using Student’s t-test or analysis of variance (ANOVA), followed by ad-hoc Tukey’s test. Probability less than 0.05 was considered as statistically significant (*p* < 0.05). Data were statistically analyzed with GraphPad Prism software (GraphPad Software Inc, San Diego, CA, USA).

## Electronic supplementary material

Supplemental information 
