# Supplementary material for: A novel peptide that improves metabolic parameters without adverse central nervous system effects
Source: Sci Rep. 2017 Nov 1;7:14781. doi: 10.1038/s41598-017-13690-9 (PMC5665932; doi:10.1038/s41598-017-13690-9)

A novel peptide that improves metabolic parameters without adverse central nervous system effects

Patrícia Reckziegel[1], William T. Festuccia[2], Luiz R. G. Britto[2], Karen L. Lopes Jang[3, 6], Carolina M. Romão[3, 6], Joel C. Heimann[3], Manoela V. Fogaça[4], Naielly S. Rodrigues[4], Nicole R. Silva[4], Francisco S. Guimarães[4], Rosangela A. S. Eichler[1], Achla Gupta[5], Ivone Gomes[5], Lakshmi A. Devi[5], Andrea S. Heimann[6*] and Emer S. Ferro[1*]

Departments of [1]Pharmacology and [2]Physiology and Biophysics, Biomedical Science Institute, and [3]Internal Medicine, School of Medicine, São Paulo, and [4]Department of Pharmacology, Ribeirão Preto Medical School, Ribeirão Preto; University of São Paulo, and [6]Proteimax Biotechnology LTDA, São Paulo, 05581-001, SP, Brazil. [5]Department of Pharmacological Sciences, Icahn School of Medicine at Mount Sinai, New York, NY 10029, USA.

Supplemental Table 1. Rational modifications of the original peptide DITADDEPLT previously described[11], driven by anti-CB1R conformational sensitive antibodies

Modifications	Peptide sequence	CB1R activity	Conclusions
None[11]	$D_1I_2T_3A_4D_5D_6E_7P_8L_9T_{10}$	Very week inverse agonist	Novel pharmacological active peptide found
T_3I (Pep19)	$D_1I_2I_3A_4D_5D_6E_7P_8L_9T_{10}$	Better inverse agonist	Position 3 is important for CB1R inverse agonism
T_3L	$D_1I_2L_3A_4D_5D_6E_7P_8L_9T_{10}$	Reduced CB1R inverse agonism, and increased CB2R inverse agonism	Leu in position 3 changes the specificity to CB2R
E_7A	$D_1I_2L_3A_4D_5D_6A_7P_8L_9T_{10}$	Week inverse agonist	Changed amino acid on position 7 is important but not critical for CB1R activity, if compared to having a hydrophobic amino acid on position 3.
T_3I – PLT	$D_1I_2L_3A_4D_5D_6E_7$	Improved inverse agonist activity	Removing three amino acids from C-terminus improved CB1R inverse agonism activity in vitro. Smallest sequence could be economically interesting; however, it was smaller with greater chance than Pep19 to cross the blood brain barrier.
P_8A	$D_1I_2I_3A_4D_5D_6E_7A_8L_9T_{10}$	Turn the peptide into agonist	Position 8 is important for CB1R activity, replacing Pro by Ala changes the peptide activity from CB1R inverse agonist to a CB1R potent agonist
Cyclic T_3I	Cyclic DIIADDEPLT	Similar inverse agonist activity to the non-cyclic peptide	No great differences were observed, could be an useful information for future *in vivo* studies

Supplemental Figure 1

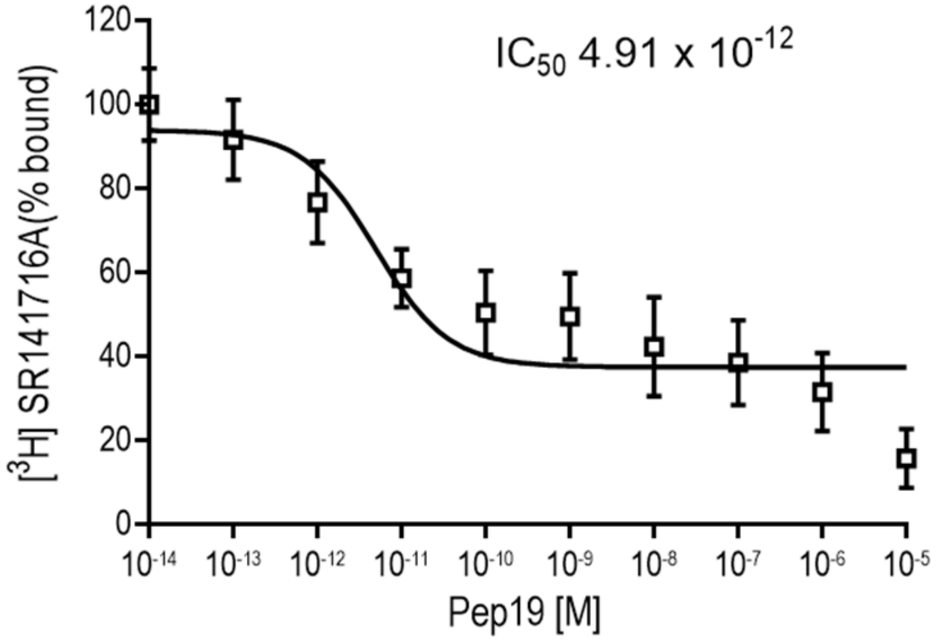

Supplemental Figure 1. Ligand binding studies with Pep19. Membranes were prepared from 3T3L-1 cells endogenously expressing CB1R, and displacement ligand binding assays were carried out as previously described[8] with minor modifications. Membranes (250 μg) were incubated with [^{3}H]SR141716A (3 nM) in 50 mM Tris-Cl, pH 7.8, containing 1 mM EGTA, 5 mM $MgCl_2$, and protease inhibitor cocktail in the presence of Pep19 (0-10 μM). Values in the absence of Pep19 were taken as 100%. Data represent mean ± SEM; *n* = 3.

Supplemental Figure 2

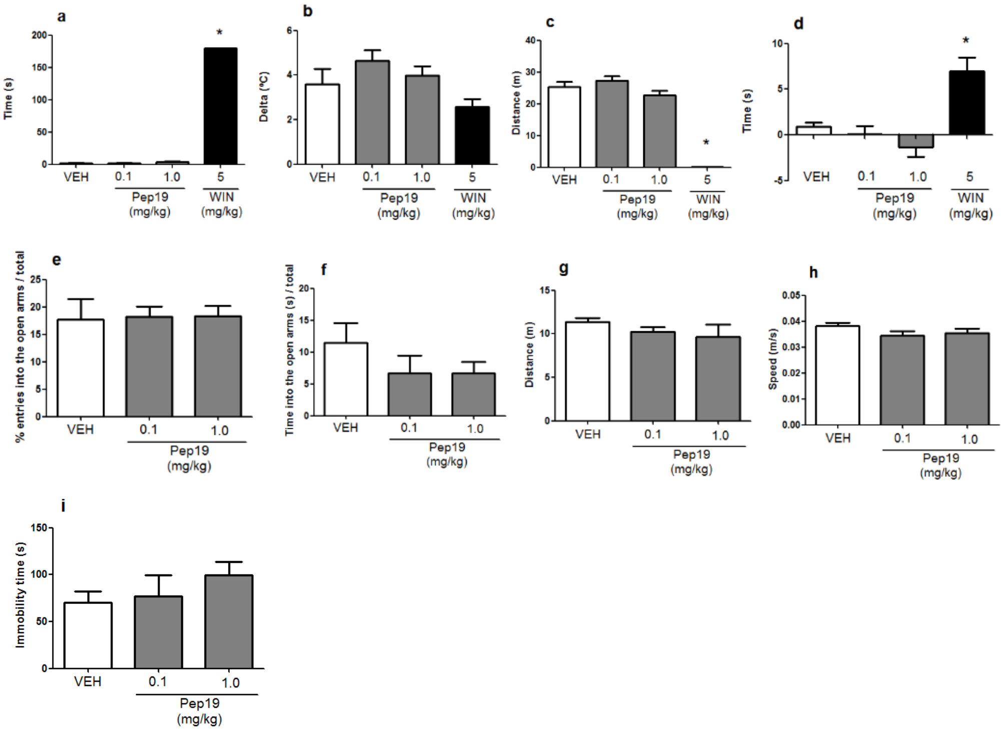

Supplemental Figure 2. *In vivo* assays showing results of chronic treatment with Pep19 *i.p.* for 10 days. To further confirm the lack of *in vivo* effects of Pep19, it was chronically administrated for 10 days, and the behavior effects were evaluated in the tetrad cannabinoid tests, consisting of **(a)** catalepsy, **(b)** tail temperature, **(c)** locomotor activity and **(d)** hot plate test. Note that no significant differences were observed between the groups treated with vehicle (saline) or Pep19 (one-way ANOVA followed by Tukey's test, n = 6/group), whereas the CB1R agonist WIN 55,212-12 affected all parameters evaluated (a-d; * $P<0.05$). In the elevated plus maze **(e, f)** the anxiety-like activity of chronic administration was evaluated following *i.p.* injections of 0.1 or 1 mg/kg of Pep19. Note that no significant differences were observed between the groups treated with vehicle or Pep19 (one-way ANOVA followed by Tukey's test, n=6-7/group). In addition, no significant differences were observed in locomotor **(g)** and speed travelled **(h)** in the elevated plus maze. In the forced swim test **(i)**, chronic administration of Pep19 *i.p.* for 10 days produced no significant differences (one-way ANOVA followed by Tukey's test, n = 6-7/ group).

Supplemental Material "Crude Western blot membranes" used to build the quantitative graphics shown on Figure 1, Panel f: Quantitative *Western* blot analysis suggested similar levels of UCP1 expression in either saline or Pep19 (600 µg/Kg) treated groups of DIO animals; (each line shown on the upper panel f, is representative of one individual animal treated with either saline or Pep19 600 µg/Kg; n = 7). All results are expressed as the means ± standard error of the mean (SEM). The statistical comparisons were performed using Student's t-test or analysis of variance (ANOVA), followed by ad-hoc Tukey's test using GraphPad Prism software.

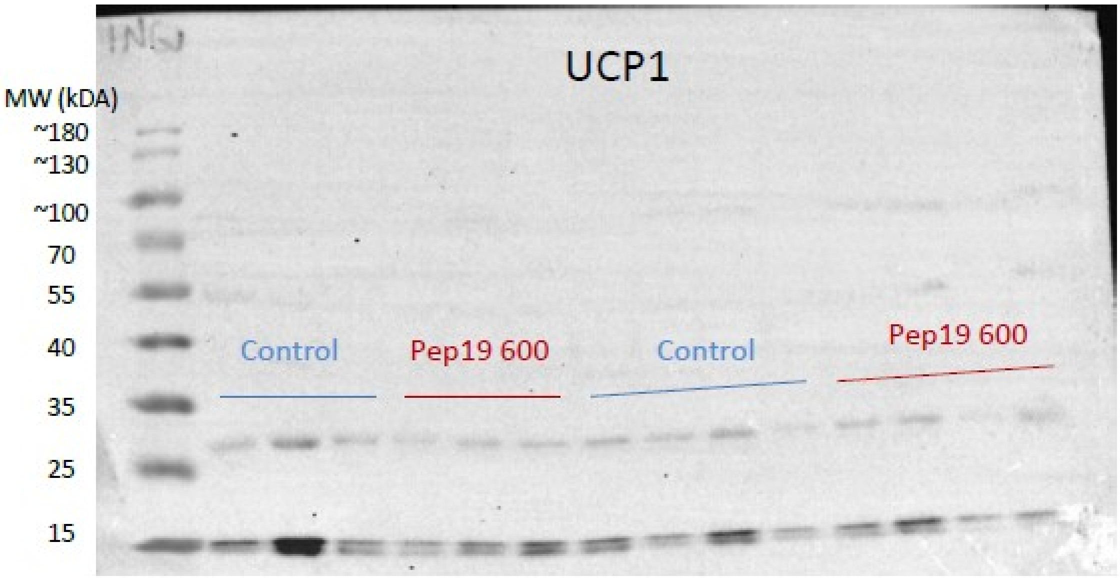

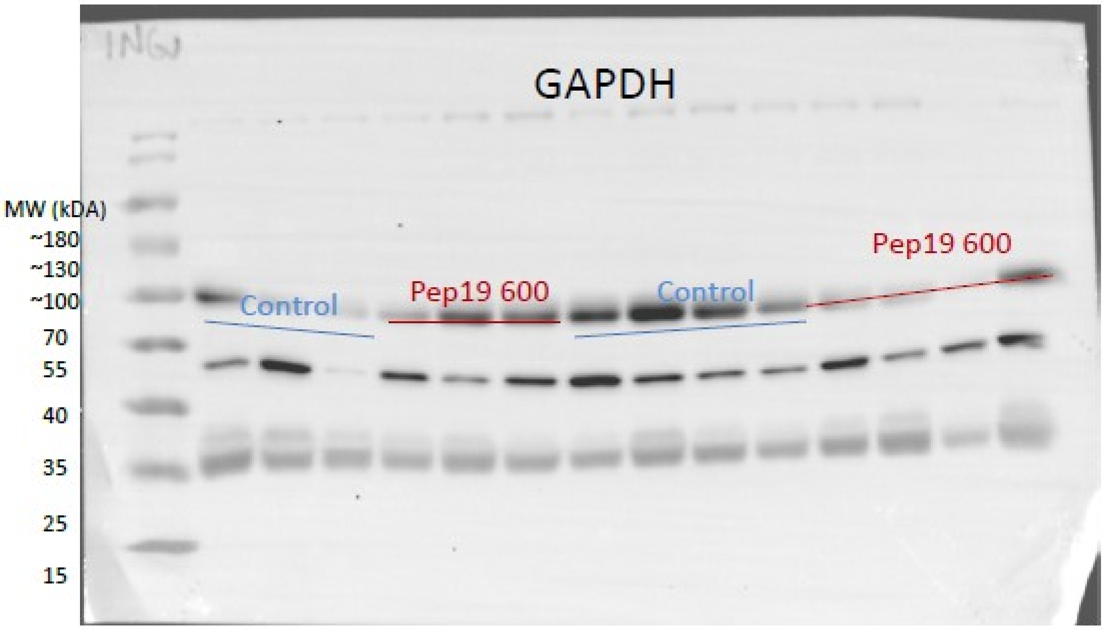

Supplemental Material "Crude Western blot membranes" used to build the quantitative graphics shown on Figure 2. Signaling pathways induced by Pep19 in 3T3-L1 adipocyte cells. **Panel a:** Cells were exposed to rosiglitazone (RSG, 5µM), or different concentrations of hemopressin (HP, 0.1-10µM) or Pep19 (0.1-10µMImaging and band intensity measurements were performed using the Odyssey imaging system (LI-COR, Lincoln, NE) according to the manufacturer's protocols. Data are representative of three independent experiments that produced similar results. Unt, cells not treated with vehicle (Veh) or peptide 19 (Pep19). The statistical comparisons were performed using Student's t-test or analysis of variance (ANOVA), followed by ad-hoc Tukey's test using GraphPad Prism software *p<0.05; **p<0.001.

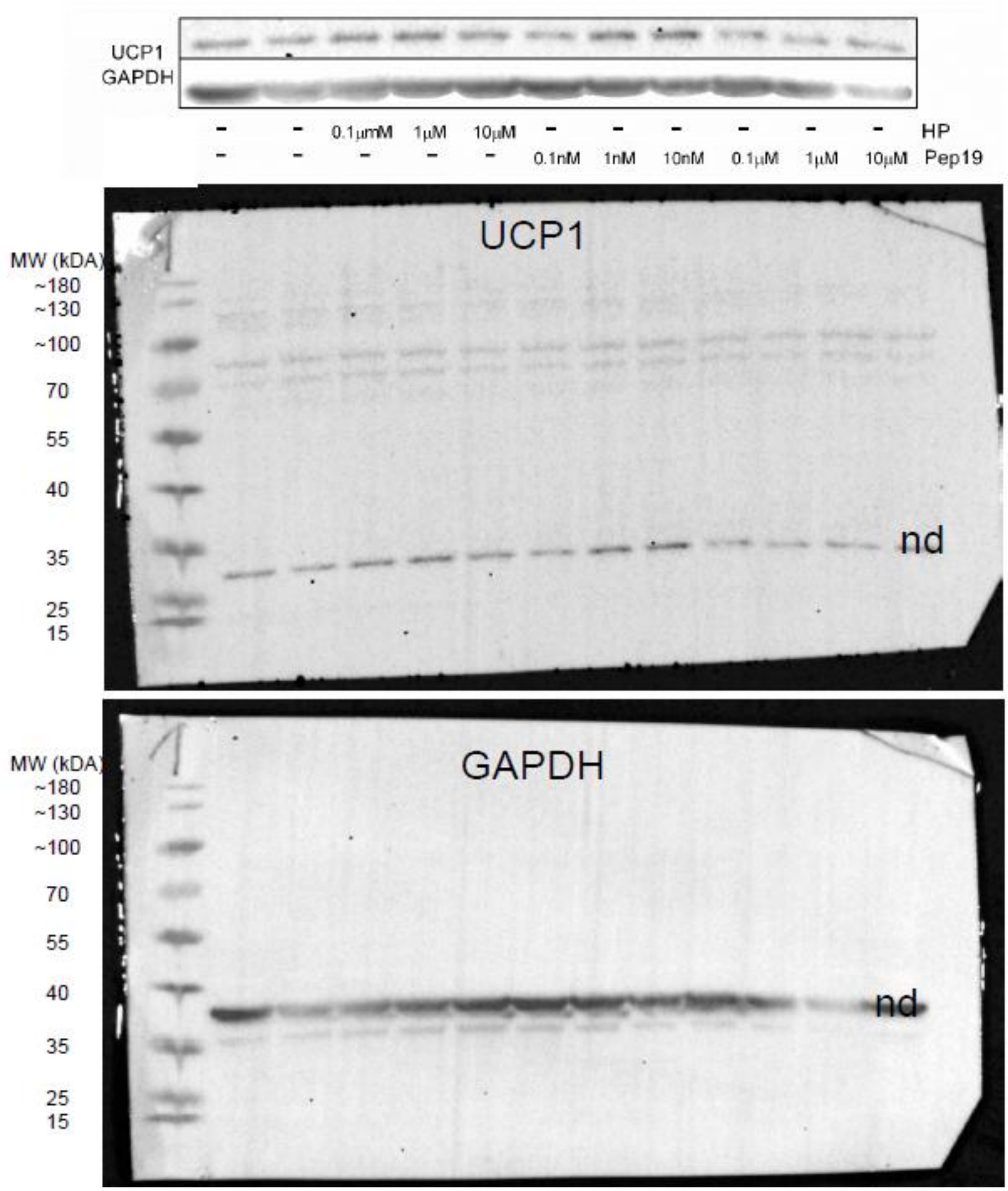

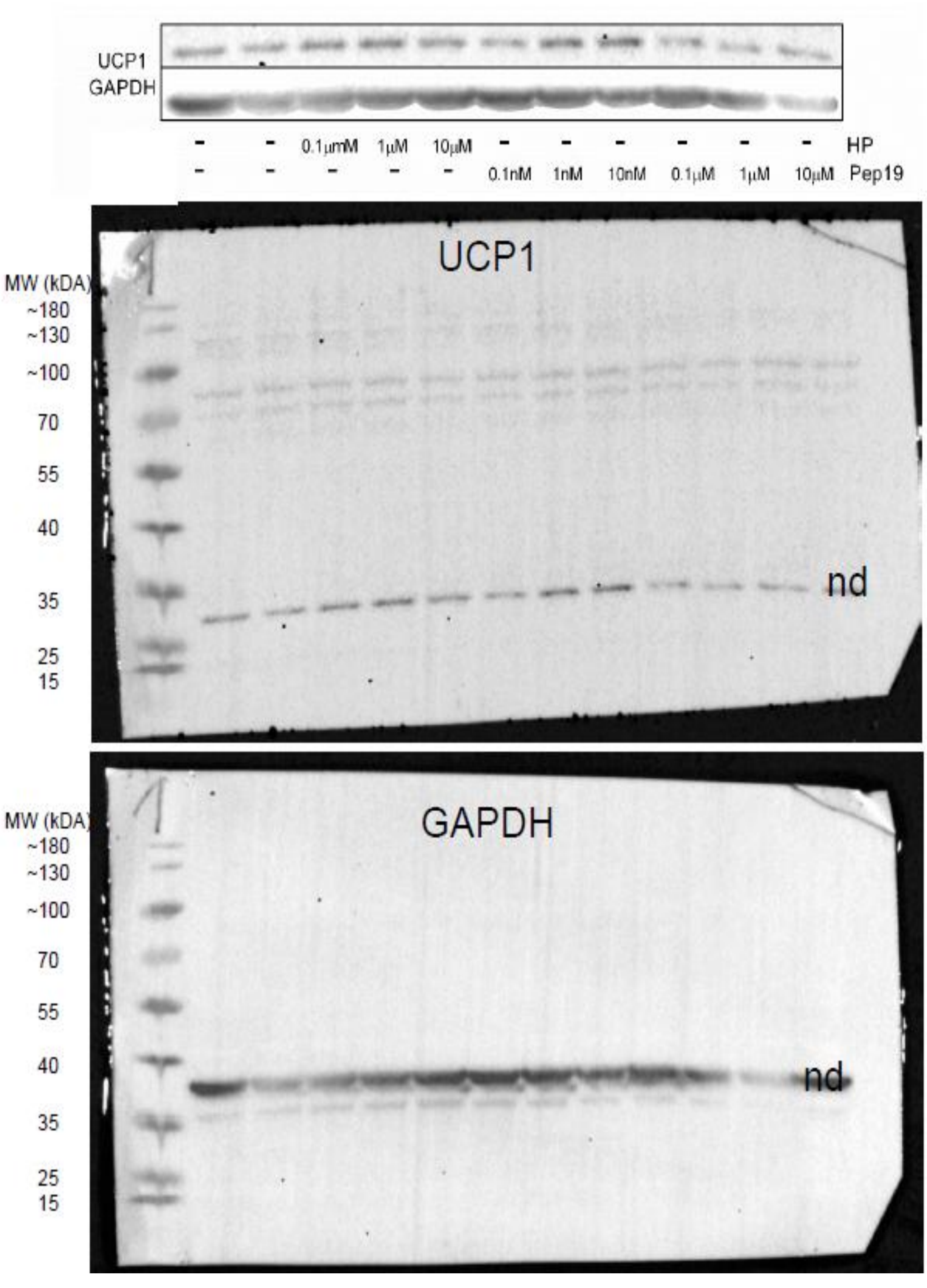

UCP1
GAPDH
- - 0.1μmM 1μM 10μM - - - - - - HP
- - - - - 0.1nM 1nM 10nM 0.1μM 1μM 10μM Pep19
UCP1
MW (kDA)
~180
~130
~100
70
55
40
35
25
15
nd
GAPDH
MW (kDA)
~180
~130
~100
70
55
40
35
25
15
nd

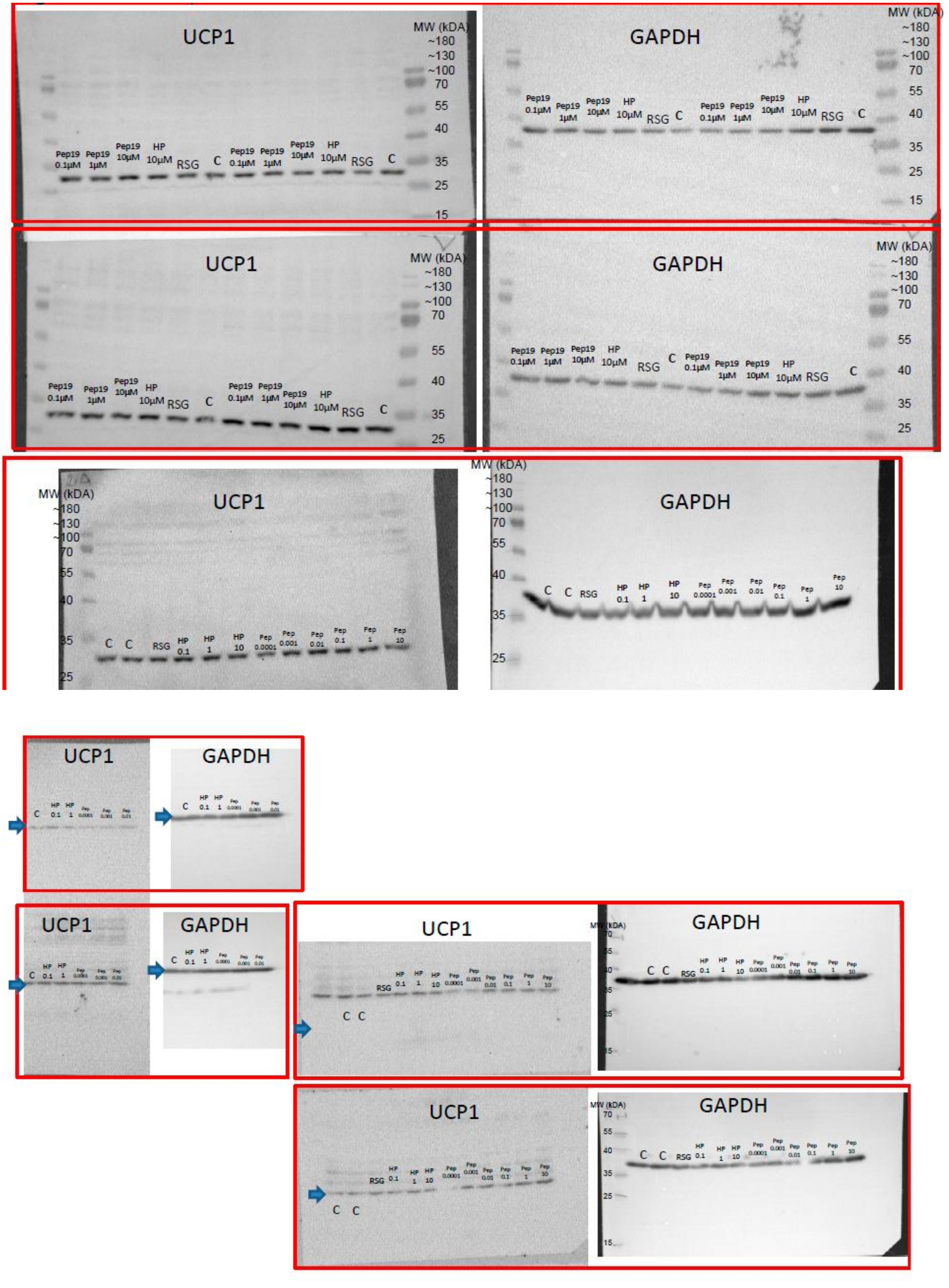
UCP1
GAPDH
MW (kDA)
~180
~130
~100
70
55
40
35
25
15
Pep19 0.1µM
Pep19 1µM
Pep19 10µM
HP 10µM
RSG
C
HP 0.1
HP 1
HP 10
Pep 0.0001
Pep 0.001
Pep 0.01
Pep 0.1
Pep 1
Pep 10

Supplemental Material "Crude Western blot membranes" used to build the quantitative graphics shown on Figure 2. Signaling pathways induced by Pep19 in 3T3-L1 adipocyte cells. **Panel b:** 3T3-L1 adipocyte cells exposed to Pep19 (1µM) for 24 h in the absence or presence of either RSG (5 µM), the CB1R agonist WIN55,212-2 (1µM), the CB1R antagonist AM251 (1µM) or the CB1R inverse agonist HP (1µM). Western blots were conducted using mouse anti-UCP1 antibodies, and anti-GAPDH antibodies were used as loading controls. Imaging and band intensity measurements were performed using the Odyssey imaging system (LI-COR, Lincoln, NE) according to the manufacturer's protocols. Data are representative of three independent experiments that produced similar results. Unt, cells not treated with vehicle (Veh) or peptide 19 (Pep19). The statistical comparisons were performed using Student's t-test or analysis of variance (ANOVA), followed by ad-hoc Tukey's test using GraphPad Prism software *p<0.05; **p<0.001.

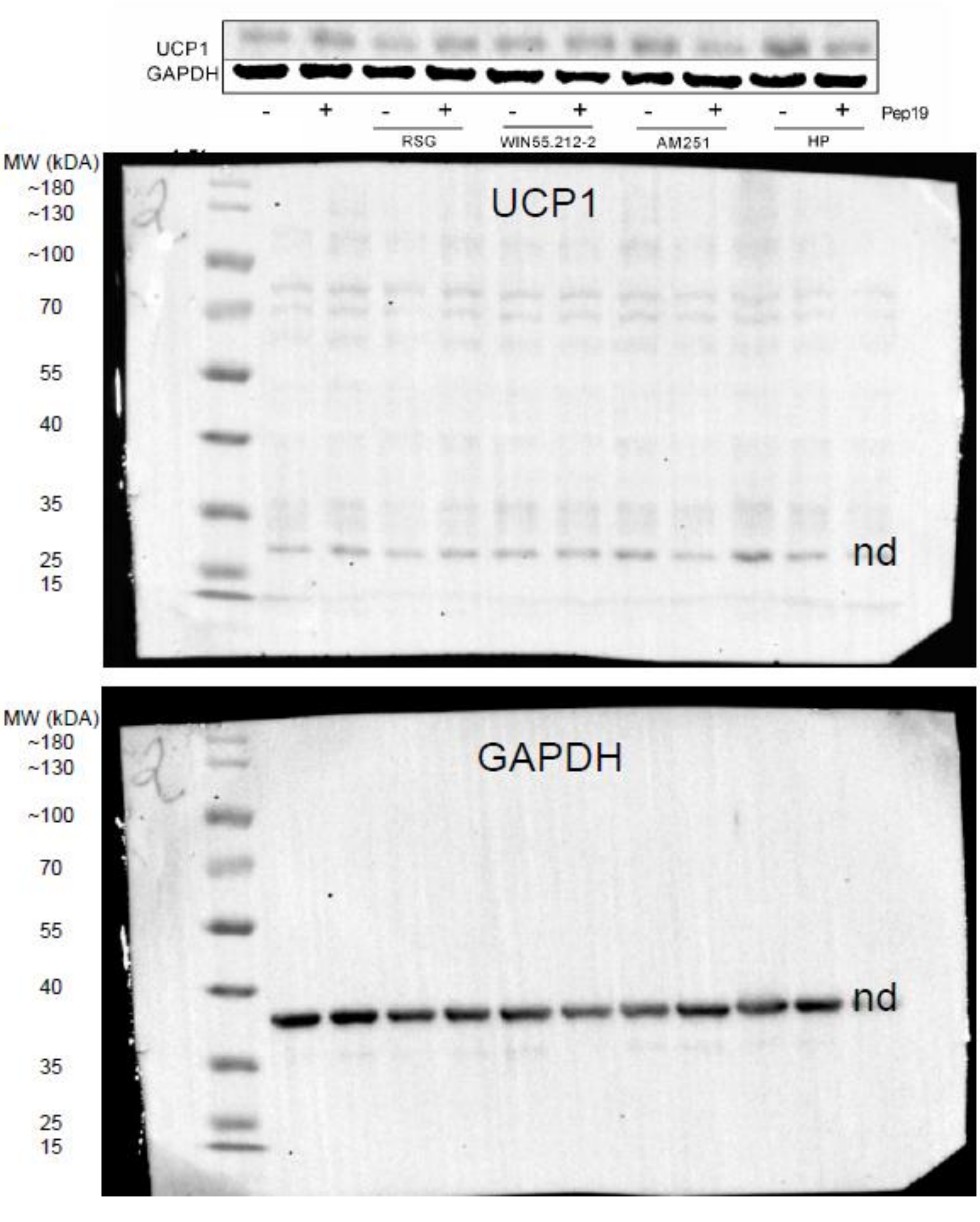

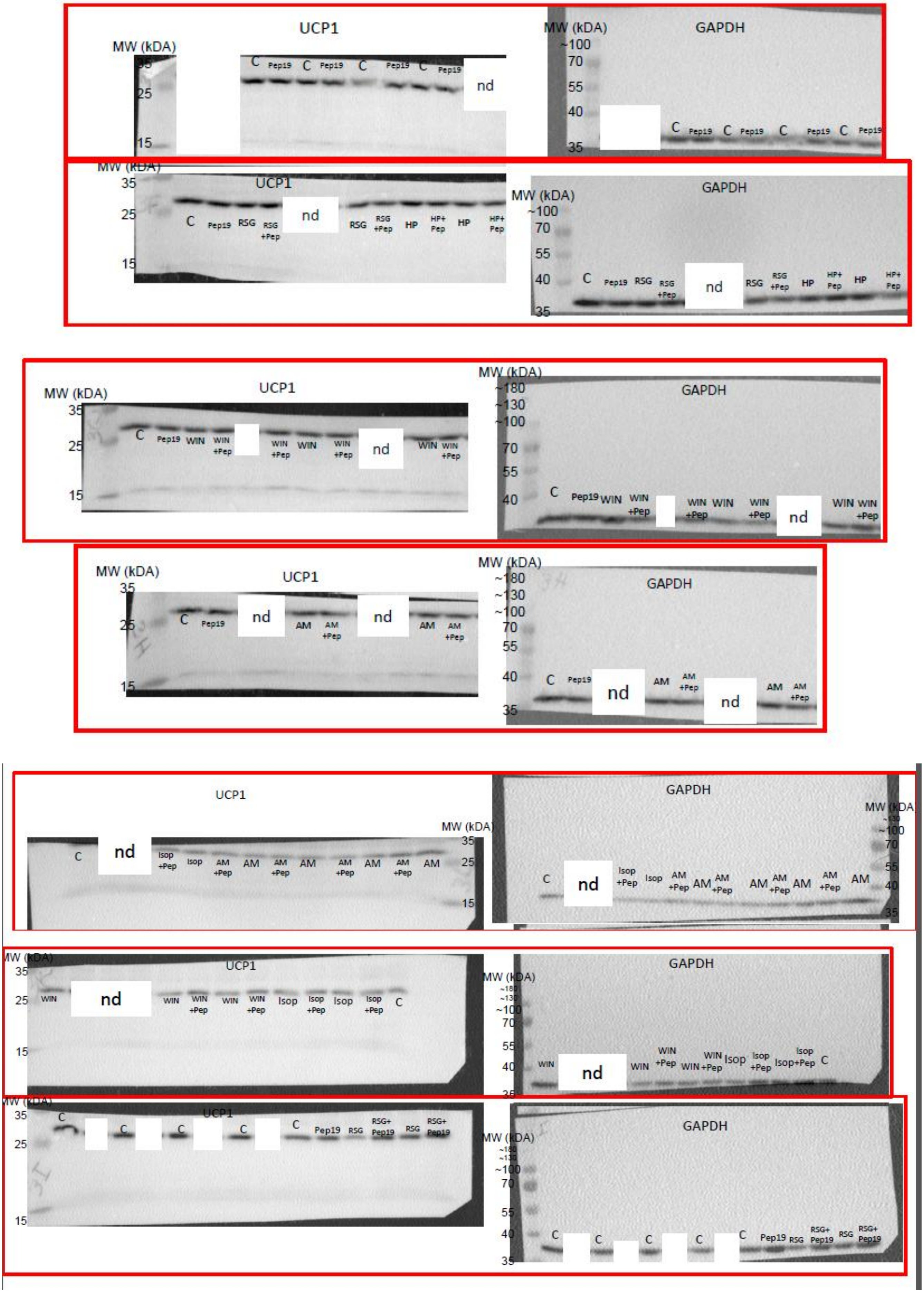

UCP1
MW (kDA)
35
25
15
C Pep19 C Pep19 C Pep19 C Pep19
nd
GAPDH
~100
70
55
40
C Pep19 RSG RSG +Pep
RSG RSG +Pep HP HP+ Pep HP HP+ Pep
~180
~130
C Pep19 WIN WIN +Pep WIN +Pep WIN WIN +Pep WIN WIN +Pep
C Pep19 AM AM +Pep AM AM +Pep
C Isop +Pep Isop AM +Pep AM AM +Pep AM AM +Pep AM AM +Pep AM
WIN WIN WIN +Pep WIN WIN +Pep Isop Isop +Pep Isop Isop +Pep C
C C C C C Pep19 RSG RSG+ Pep19 RSG RSG+ Pep19

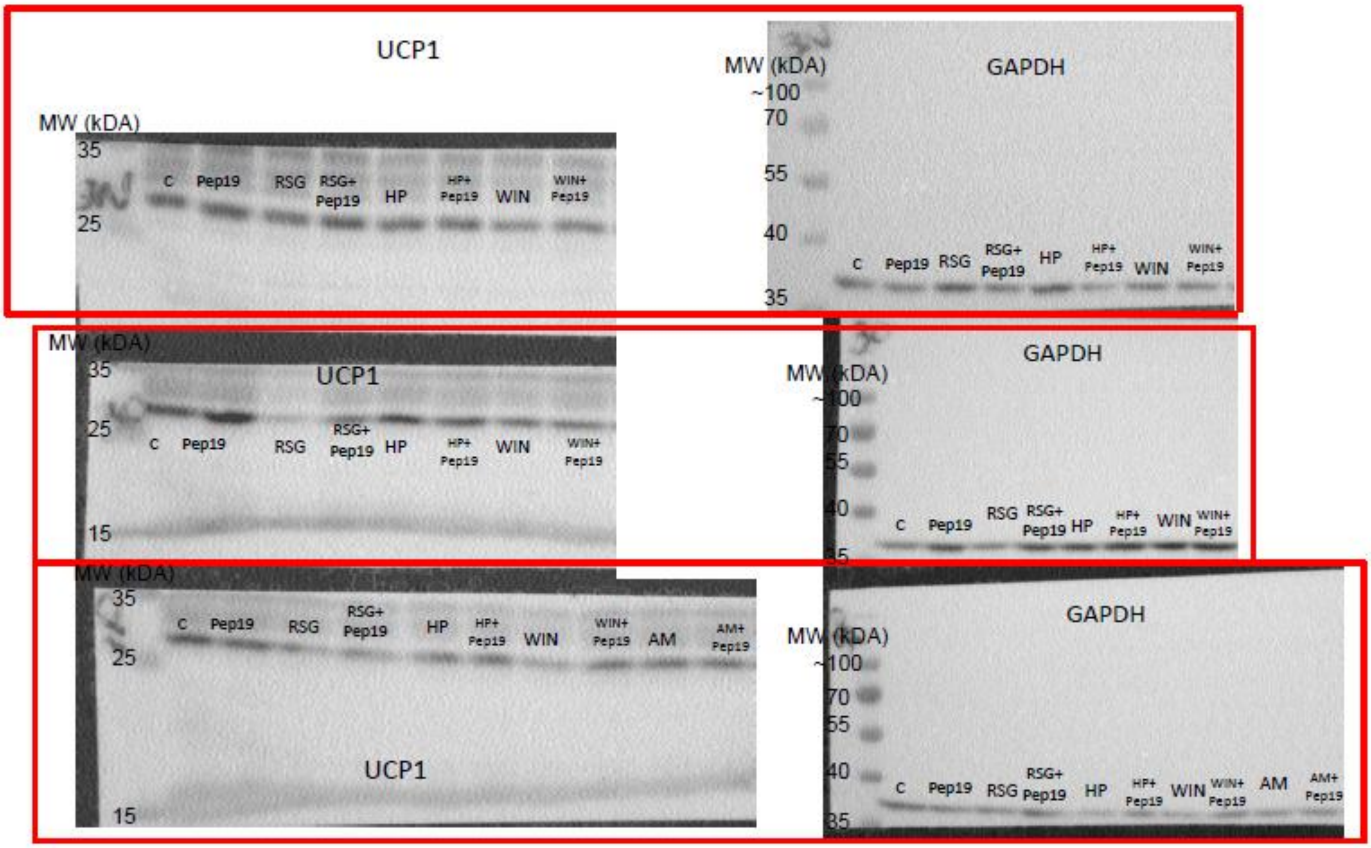
UCP1
MW (kDA)
35
25
C Pep19 RSG RSG+ Pep19 HP HP+ Pep19 WIN WIN+ Pep19
GAPDH
MW (kDA)
~100
70
55
40
35
C Pep19 RSG RSG+ Pep19 HP HP+ Pep19 WIN WIN+ Pep19
MW (kDA)
35
UCP1
25
C Pep19 RSG RSG+ Pep19 HP HP+ Pep19 WIN WIN+ Pep19
15
GAPDH
MW (kDA)
~100
70
55
40
35
C Pep19 RSG RSG+ Pep19 HP HP+ Pep19 WIN WIN+ Pep19
MW (kDA)
35
C Pep19 RSG RSG+ Pep19 HP HP+ Pep19 WIN WIN+ Pep19 AM AM+ Pep19
25
UCP1
15
GAPDH
MW (kDA)
~100
70
55
40
35
C Pep19 RSG RSG+ Pep19 HP HP+ Pep19 WIN WIN+ Pep19 AM AM+ Pep19

Supplemental Material "Crude Western blot membranes" used to build the quantitative graphics shown on Figure 2. Signaling pathways induced by Pep19 in 3T3-L1 adipocyte cells. **Panels c and d:** 3T3-L1 cells were starved for 16h in serum-free medium prior to stimulation (vehicle or Pep19, 1 μM) for the indicated time period. Western blots were carried out using: **Panel c,** mouse monoclonal anti-phosphoERK1/2, and rabbit polyclonal anti-total ERK1/2; **Panel d,** rabbit phospho-AKT S473 (anti-pAKT, S473) and mouse monoclonal anti-tubulin antibodies. Imaging and band intensity measurements were performed using the Odyssey imaging system (LI-COR, Lincoln, NE) according to the manufacturer's protocols. Data are representative of three independent experiments that produced similar results. Unt, cells not treated with vehicle (Veh) or peptide 19 (Pep19). The statistical comparisons were performed using Student's t-test or analysis of variance (ANOVA), followed by ad-hoc Tukey's test using GraphPad Prism software *p<0.05; **p<0.001.

Panel c

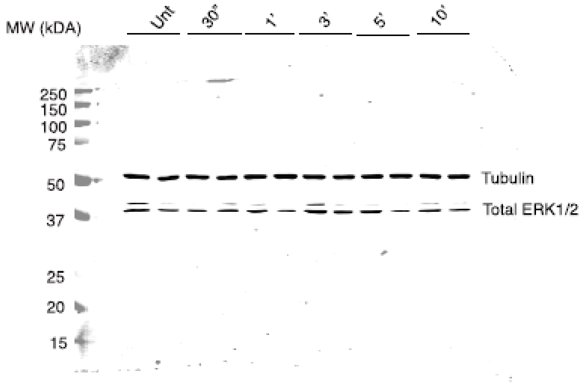

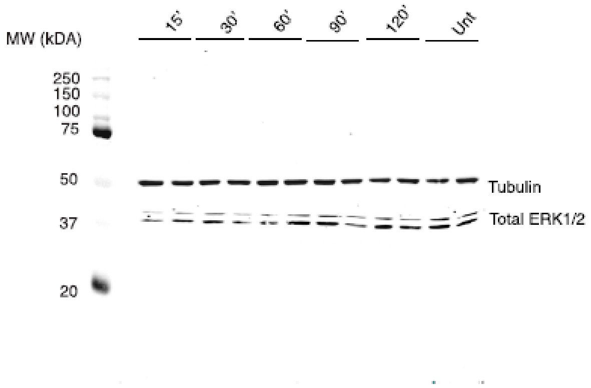

Panel d

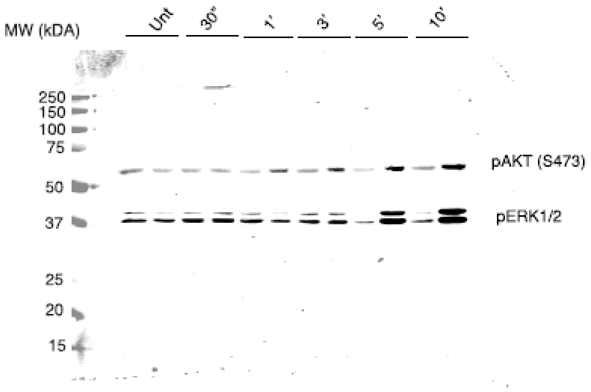

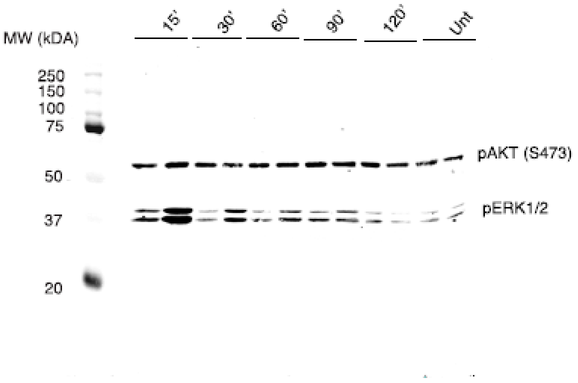

Supplement: Supplementary file 1 — Supplemental information [file 41598_2017_13690_MOESM1_ESM.pdf]
